# Population genetic and phytochemical dataset of *Saraca asoca*: A traditionally important medicinal tree

**DOI:** 10.1016/j.dib.2019.104173

**Published:** 2019-06-25

**Authors:** Satisha Hegde, Sandeep Ramchandra Pai, Rasika M. Bhagwat, Archana Saini, Poonam Kanwar Rathore, Sunil Satyappa Jalalpure, Harsha Vasudev Hegde, Attayoor Purushottaman Sugunan, Vidya S. Gupta, Sanjiva D. Kholkute, Subarna Roy

**Affiliations:** a ICMR – National Institute of Traditional Medicine, Indian Council of Medical Research, Department of Health Research, Govt. of India, Belagavi, Karnataka, 590010, India; b KLE Academy of Higher Education and Research (Deemed-to-be-University), Dr. Prabhakar Kore Basic Science Research Center, Belagavi, Karnataka, 590010, India; c Amity Institute of Biotechnology, Amity University, Mumbai - Pune Expressway, Bhatan, Post – Somathne, Panvel, Mumbai, Maharashtra, 410206, India; d Plant Molecular Biology Group, Division of Biochemical Sciences, CSIR - National Chemical Laboratory, Pune, Maharashtra, 411008, India; e Department of Pharmacognosy and Phytochemistry, College of Pharmacy, Belagavi, KLE Academy of Higher Education and Research (Deemed-to-be-University), Belagavi, Karnataka, 590010, India; f Epidemiology Division, RMRC-NIE-LRU, National Institute of Epidemiology, Indian Council of Medical Research, Department of Health Research, Govt. of India, Chennai, Tamil Nadu, 600 077, India

**Keywords:** Chemical profiling, Conservation, Genetic diversity, Population genetics, Statistical analysis, Western ghats

## Abstract

The data presented in this article is in support of the research paper “Genetic and phytochemical investigations for understanding population variability of the medicinally important tree *Saraca asoca* to help develop conservation strategies” Hegde et al., 2018. This article provides PCR based Inter-Simple Sequence Repeat (ISSR) and HPLC datasets of 106 individual samples of *Saraca asoca* collected from various geographical ranges of the Western Ghats of India. The ISSR data includes information on genetic diversity and images of population structures generated through amplified DNA products from samples of *Saraca asoca* leaf. Phytochemical data obtained from HPLC includes concentration (mg/g) of gallic acid (GA), catechin (CAT), and epicatechin (EPI). The data also presents information obtained from various statistical analysis *viz.* standard error of the mean values, distribution variables, prediction accuracy, and multiple logistic regression analysis.

**Table:** Specifications table

Subject area	*Biology*
More specific subject area	*Molecular Biology and Phytochemistry*
Type of data	*Table, graph, figure*
How data was acquired	*PCR (Mastercycler® Nexus, Eppendorf, Germany) and HPLC (Shimadzu chromatographic system, Model no. LC-20AD)*
Data format	*Analysed and statistical data*
Experimental factors	*Saraca asoca leaf and bark samples were collected from 106 accessions of 11 populations. For genetic analysis portions of leaf samples from each accession were stored at -80°C. Remaining leaf and bark samples were shade dried and powdered before processing.*
Experimental features	*DNA isolated from the leaf samples were used for ISSR fingerprinting. Twenty primers were used for ISSR assay. Dried leaf and bark powder of 5g each was used for extraction by washing with petroleum ether followed by methanol: water (70:30) extraction in triplicate and evaporated to dryness. These extracts were further used for HPLC analysis.*
Data source location	*Western Ghats, India*
Data accessibility	*Data available within this article. Supplementary data associated with this article can be found in the online version at*https://doi.org/10.1016/j.phytochem.2018.08.016
Related research article	*S. Hegde, S.R. Pai, R.M. Bhagwat, A. Saini, P.K. Rathore, S.S. Jalalpure, H.V. Hegde, A.P. Sugunan, V.S. Gupta, S.D. Kholkute, S. Roy, Genetic and phytochemical investigations for understanding population variability of the medicinally important tree Saraca asoca to help develop conservation strategies, Phytochemistry 156 (2018) 43–54.*

**Table:** 

**Value of data** • The data presented here will provide information on the genetic and phytochemical profiles of a 106 accessions of *S. asoca* in various parts of the Western Ghats which is useful to understand population genetics and phytochemical variability (with respect to selected major compounds) of this important medicinal tree species. • The data could be used in future investigations of *S. asoca* and will help develop its conservation strategies.

## 1 Data

**Fig. 1:**
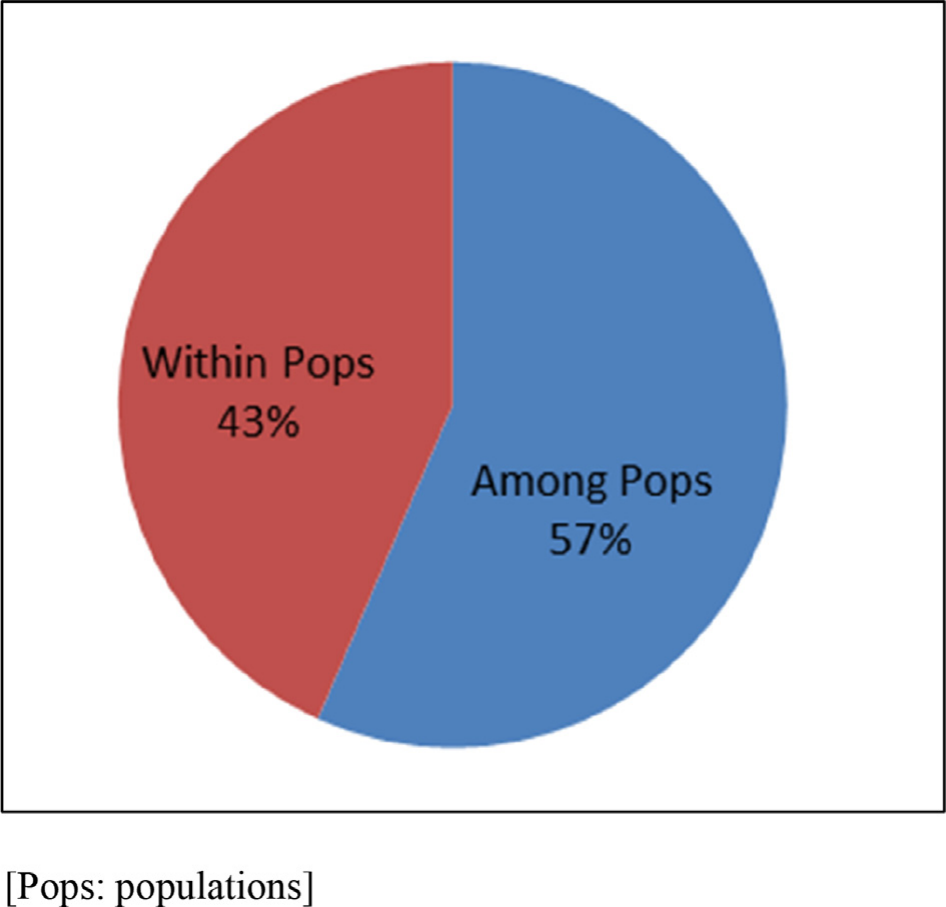
Percentage of molecular variance of 106 individuals from 11 populations of *S. asoca.*

**Fig. 2:**
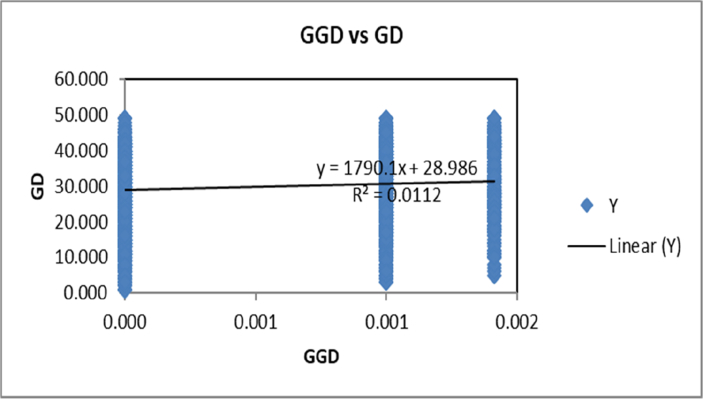
Relationship between genetic distance and geographical distances of 106 individuals from 11 populations of *S. asoca* using ISSR markers by mantel test (GD: genetic distance; GGD: geographical distance).

**Fig. 3:**
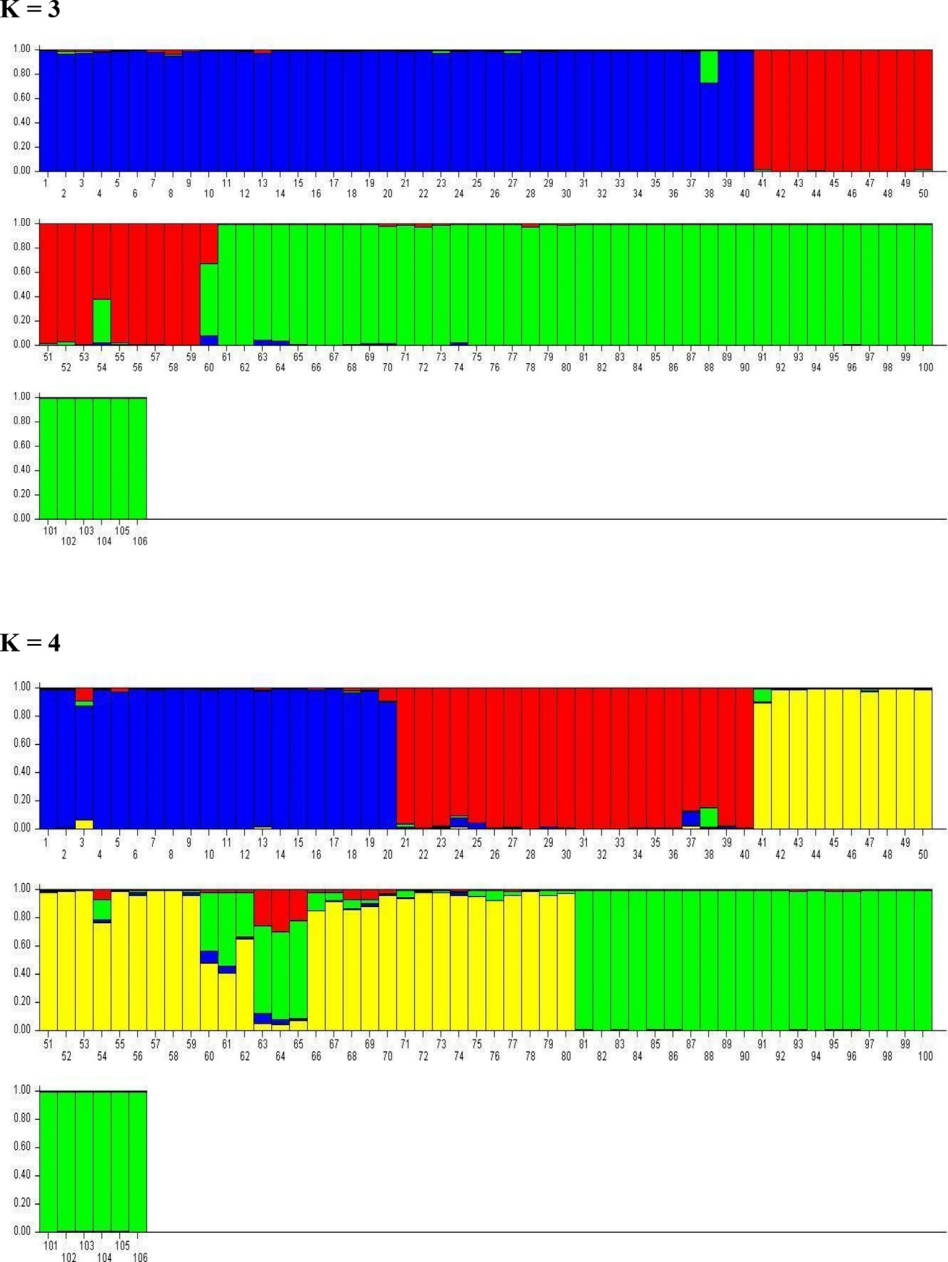

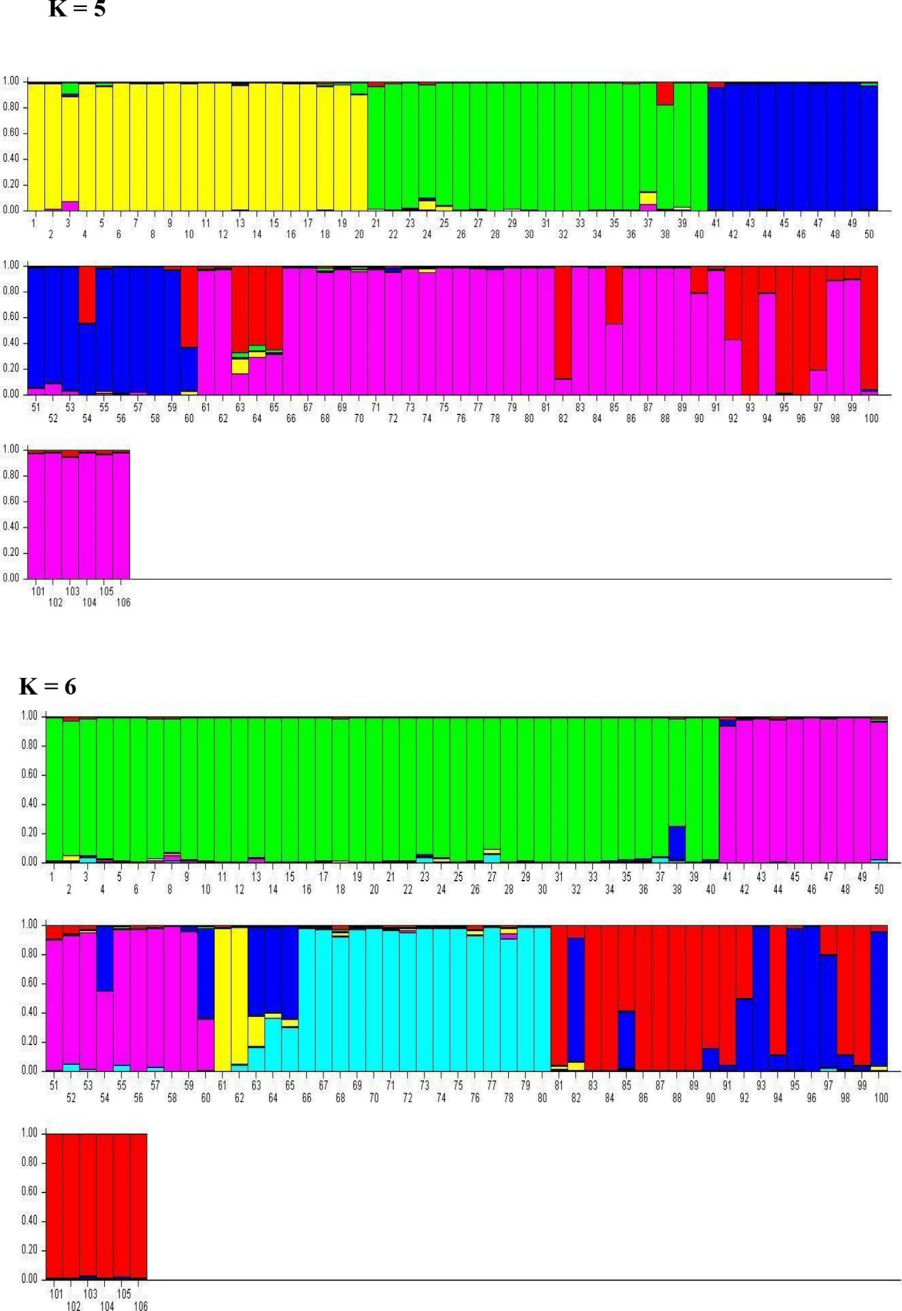
Population structure of 106 samples of *S. asoca* collected from 11 populations, by admixture analysis (K = 3; K = 4; K = 5; K = 6); each individuals is represented by vertical line (sample number: 1–40 = TIL, AMG, GHA, DEV & 41–106: JAD, KOD, BIL, THI, BON, HEG, MAD).

**Fig. 4:**
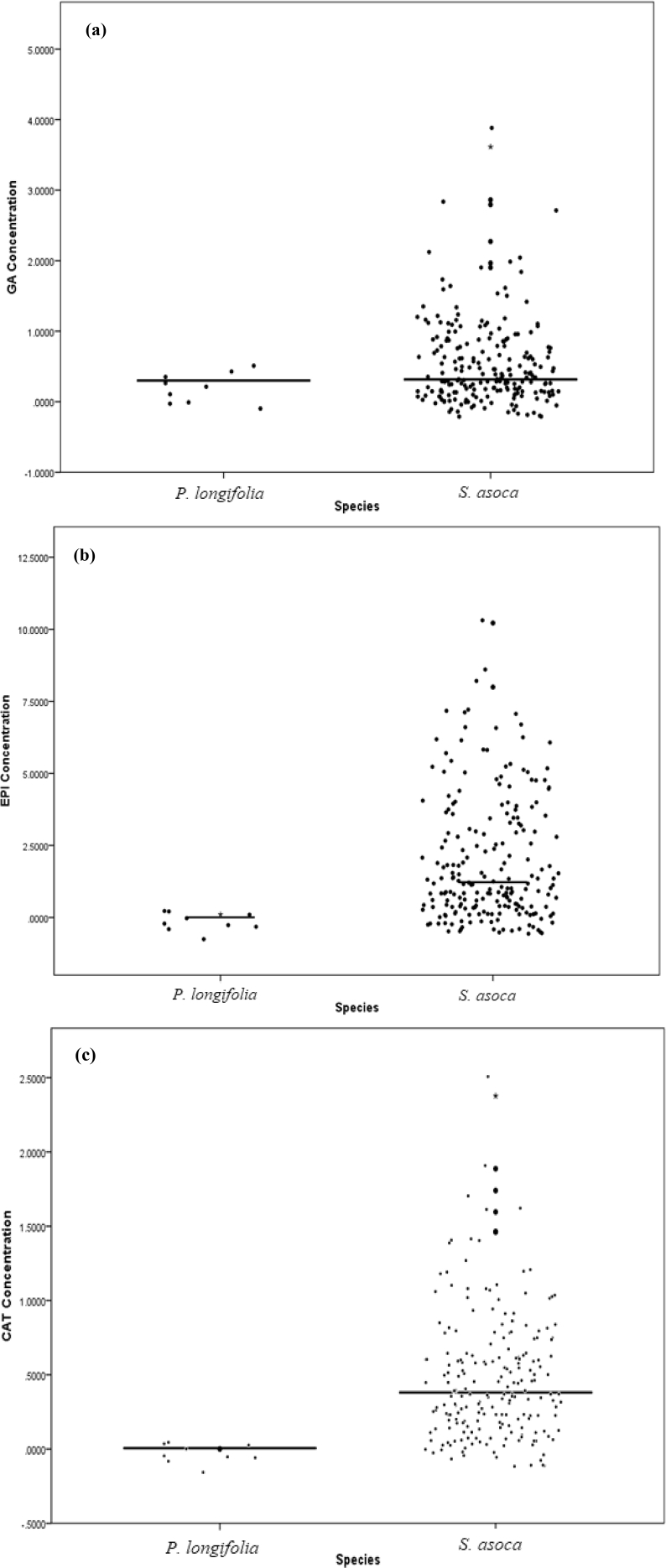
(a): Distribution of *Saraca asoca* and *Polyalthia longifolia* (control) samples by gallic acid (GA) concentration, horizontal line corresponds to median value (see Table 2(A), Table 2(B)). (b): Distribution of *Saraca asoca* and *Polyalthia longifolia* (control) samples by epicatechin (EPI) concentration, horizontal line corresponds to median value (see Table 2(A), Table 2(B)). (c): Distribution of *S. asoca* and *Polyalthia longifolia* (control) samples by catechin (CAT) concentration, horizontal line corresponds to median value (see Table 2(A), Table 2(B)).

**Fig. 5:**
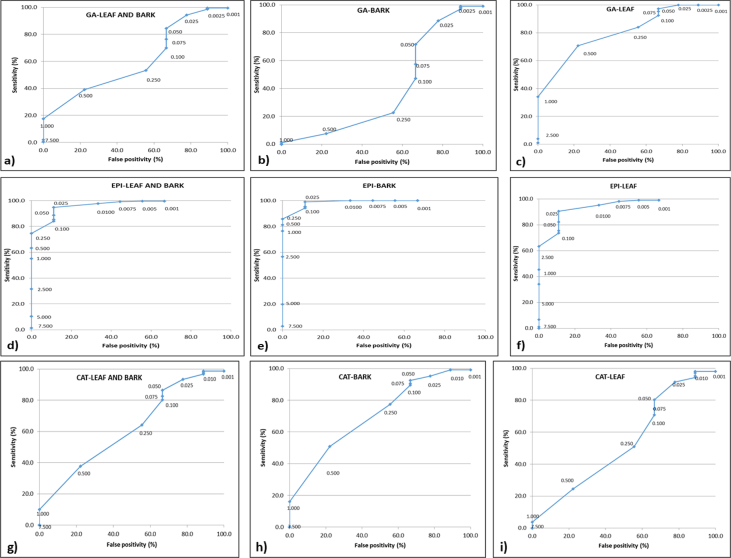
Receiver Operating Characteristic (ROC) plots of gallic acid (GA), epicatechin (EPI) and catechin (CAT) contents in leaf and bark of 106 individuals from 11 populations of *S. asoca.* [ROC plots of **a)** GA leaf and bark; **b)** GA bark; **c)** GA leaf; **d)** EPI leaf and bark **e)** EPI bark; **f)** EPI leaf; **g)** CAT leaf and bark; **h)** CAT bark; **i)** CAT leaf].

**Fig. 6:**
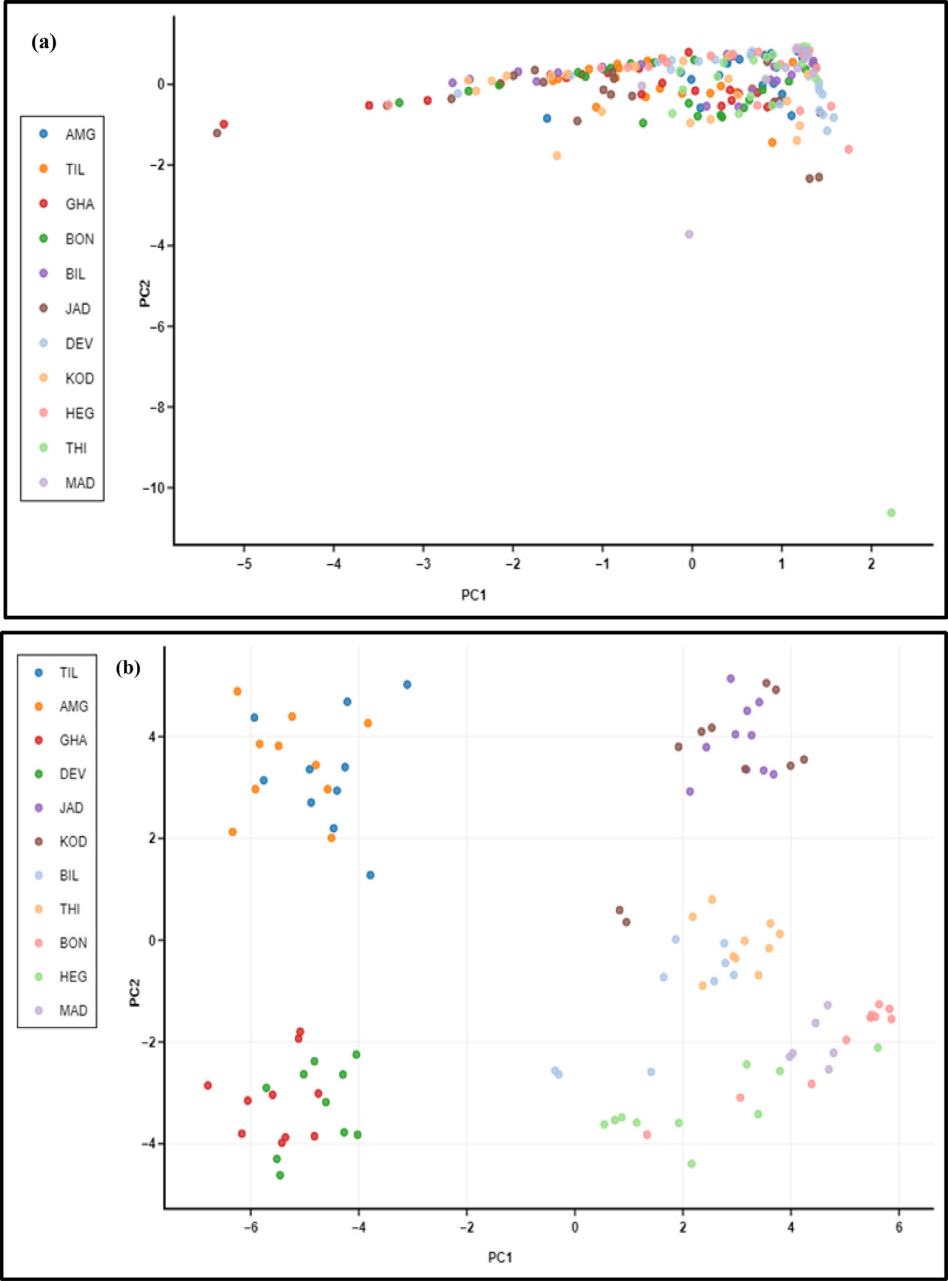
Principal Component Analysis (PCA) of 106 individuals from 11 populations of *S. asoca.* (a) Combined bark and leaf PCA for gallic acid (GA), epicatechin (EPI) and catechin (CAT) contents (mg/g). (b) ISSR based PCA for 11 populations of 106 samples of *S. asoca.*

**Fig. 7:**
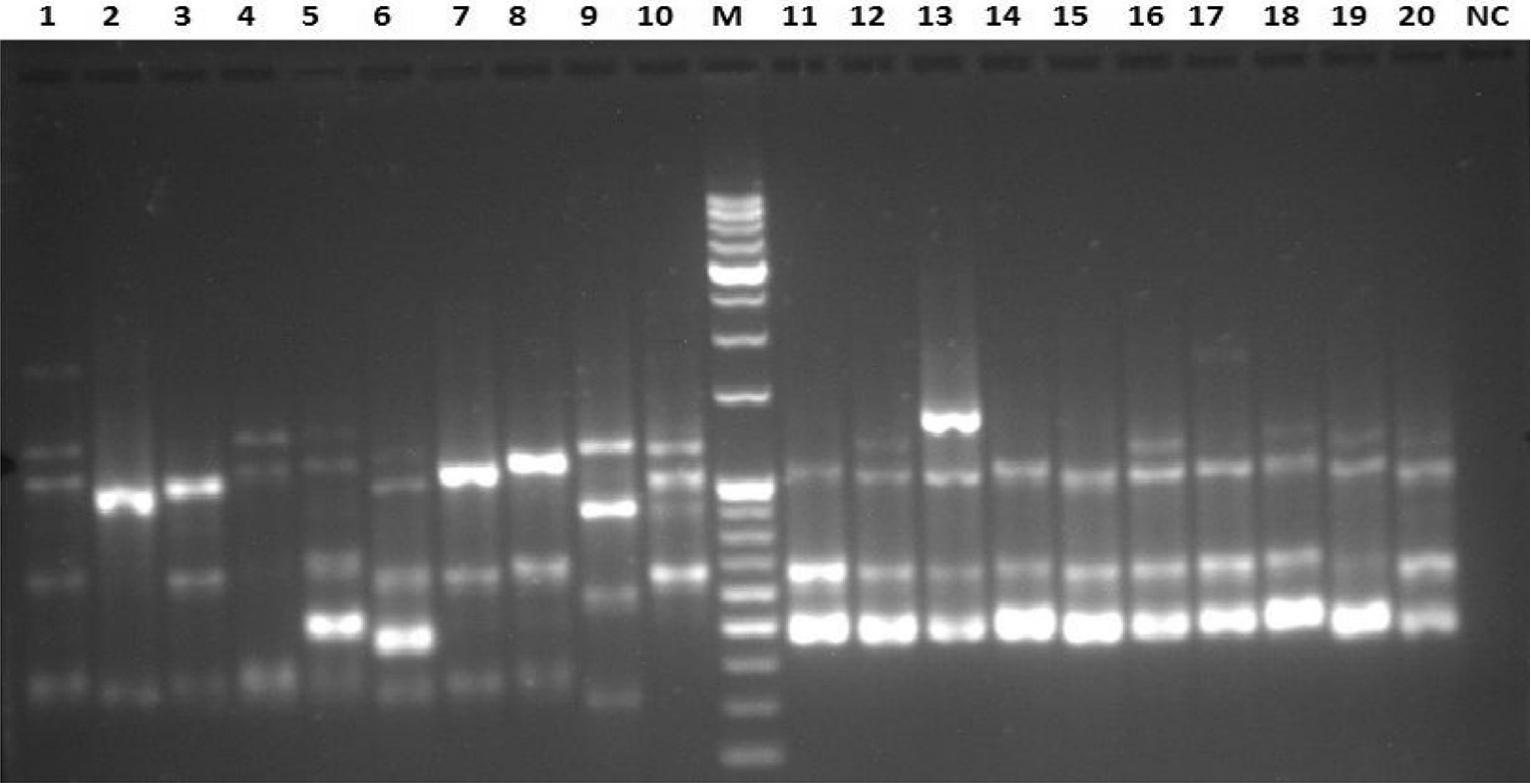
ISSR fingerprints of *S. asoca* with primer UBC814 (M: 500+100bp Mol. wt. markers, NC: Negative control, Lane 1–10:GHA and 11–20:DEV samples, sorted according to the population code from Table 5[1]).

The data presented here was the basis of the research article by Hegde et al. [1]. We present the data of seven figures and seven tables related to the research article Hegde et al. [1]. The first figure (Fig. 1) presents the percentage of molecular variance of 106 individuals of *Saraca asoca* collected from 11 populations. The second figure (Fig. 2) represents the relationship between genetic distances and geographical distances of the above samples using ISSR markers by Mantel test. The third figure (Fig. 3) presents population structure of samples, by admixture analysis. These datasets were obtained after the testing of 20 primers and selecting only those that showed reproducible bands upon repetition of the assays. Based on the presence (1) and absence (0) of bands, the gel profiles were scored. Various multivariate analyses were carried out on the binary data thus obtained, applying statistical tools to obtain the results. The fourth figure (Fig. 4) compares *S. asoca* with one of its adulterants/substituents *Polyalthia longifolia* and it provides information on the distribution of these two species with reference to concentrations of three phytochemical constituents used as markers in the study viz., a) distribution of *S. asoca* and *P. longifolia* samples by gallic acid (GA) concentration, b) distribution of *S. asoca* and *P. longifolia* samples by epicatechin (EPI) concentration and c) distribution of *S. asoca* and *P. longifolia* samples by catechin (CAT) concentration. The data presented here has been obtained after quantification and analysis of GA, CAT and EPI from the *S. asoca* leaf and bark extracts using HPLC while those from *P. longifolia* has been obtained from previous literature on *P. longifolia* [2]. The fifth figure (Fig. 5) presents Receiver Operating Characteristic (ROC) plots of GA, EPI and CAT contents in leaf and bark of 106 individuals from 11 populations of *S. asoca.* The dataset has been obtained after quantification and analysis of GA, CAT and EPI from *S. asoca* leaf and bark extracts (mg/g) using HPLC. The sixth figure (Fig. 6) presents Principal Component Analysis (PCA) of the *S. asoca* samples, a) with respect to combined GA, EPI and CAT contents in bark and leaf or and b) with respect to ISSR based genetic markers. The dataset has been obtained after analysis of quantities of GA, CAT and EPI and from binary data obtained after scoring of DNA fingerprints from the *S. asoca* respectively. The seventh figure (Fig. 7) presents ISSR fingerprints of *S. asoca* with primer UBC814. This data has been acquired after electrophoresis of amplified PCR products in agarose gels and photographed using gel documentation system (Syngene, UK).

The first table (Table 1) presents details about the primers used in ISSR assays and the amplification profiles in 11 populations of *S. asoca.* These datasets were obtained after testing of 20 primers and selecting only those primers that consistently produced reproducible bands in at least three independent repeat assays. The data was acquired from binary data scored using the fingerprints obtained from *S. asoca* samples with presence and absence of individual bands taken as 1 and 0 respectively. The second (Table 2(A), Table 2(B), Table 2(C), Table 2(D)) and third tables (Table 3(A), Table 3(B), Table 3(C), Table 3(D)) present contents of GA, CAT and EPI in *S. asoca* samples quantified (mg/g) using HPLC technique. The fourth table (Table 4) presents standard error of the mean (SEM) of chemical constituent GA, CAT and EPI. The fifth table (Table 5) presents the variations in the total chemical constituent (mg/g) within the 11 populations of *S. asoca.* The second, third, fourth, and fifth tables show data acquired from HPLC assay with further statistical analysis. The sixth table (Table 6) presents the information on the ISSR markers that are highly associated with (≥75th percentile) concentration of phytochemicals in 11 *S. asoca* populations. The seventh table (Table 7) presents prediction accuracy of models for phytochemical content (≥75th percentile = high, else = Low) in 11 *S. asoca* populations. These data (Table 6, Table 7) were obtained from multiple regression analysis of both HPLC data and ISSR based binary data obtained from 106 accessions of *S. asoca*.

**Table 1:** Details about amplification profiles of ISSR markers in 11 populations of *S. asoca*.

Sl. No.	Primer name	Primer Sequence (5′ to 3′)	Number of loci	PIC	MI
1	UBC814	(CT)_8_A	12	0.215	0.700
2	UBC815	(CT)_8_G	6	0.495	1.304
3	UBC834	(AG)_8_YT∗	11	0.388	1.877
4	UBC841	(GA)_8_YC∗	10	0.474	2.116
5	UBC845	(CT)_8_RG∗∗	13	0.306	1.010
6	UBC855	(AC)_8_YT∗	12	0.317	1.103
7	UBC880	(GGAGA)_3_	10	0.499	2.496
Total			74	2.697	10.609
Average			10.57	0.385	1.515

∗ Y= C+T; ∗∗R=A+G; PIC: Polymorphic Information Content; MI: Marker Index.

**Table 2(A):** Contents GA, CAT and EPI (mg/g) in bark samples of *S. asoca* from AMG, DEV and THI.

Sample No.	AMG			DEV			THI		
	GA	CAT	EPI	GA	CAT	EPI	GA	CAT	EPI
1	0.669±0.033	0.296±0.014	0.148±0.007	0.014±0.001	0.222±0.011	1.397±0.069	0.025±0.001	0.016±0.001	0.075±0.003
2	0.816±0.040	1.370±0.068	2.379±0.118	0.048±0.002	0.479±0.023	1.252±0.062	0.155±0.007	0.274±0.013	0.543±0.027
3	0.359±0.017	0.195±0.009	0.330±0.016	0.228±0.011	1.267±0.063	5.412±0.270	0.596±0.029	0.634±0.031	1.406±0.070
4	0.232±0.011	0.065±0.003	0.033±0.001	0.154±0.007	0.609±0.030	1.609±0.080	0.003±0.001	0.022±0.001	0.038±0.001
5	0.942±0.047	0.807±0.040	0.213±0.010	0.063±0.003	0.387±0.019	1.177±0.058	0.116±0.005	0.235±0.011	0.270±0.013
6	0.329±0.016	0.778±0.038	0.208±0.010	0.045±0.002	0.364±0.018	2.714±0.135	0.081±0.004	0.203±0.010	0.199±0.009
7	0.698±0.034	0.306±0.015	0.494±0.024	0.058±0.002	0.403±0.020	2.988±0.149	0.153±0.007	0.720±0.036	0.744±0.037
8	0.126±0.006	0.473±0.023	0.392±0.019	0.091±0.004	0.872±0.043	3.555±0.177	0.097±0.004	0.495±0.024	0.389±0.019
9	1.396±0.069	0.267±0.013	0.243±0.012	0.054±0.002	0.256±0.012	0.662±0.033	0.430±0.021	0.392±0.019	1.346±0.067
10	0.945±0.047	0.196±0.009	0.576±0.028	0.014±0.001	0.122±0.006	1.254±0.062	0.404±0.020	ND	1.417±0.070

GA: gallic acid; CAT: catachin; EPI: epicatachin; ND: not detected: AMG: Amgaon; DEV: Devimane Ghat; THI: Thirthahalli.

**Table 2(B):** Contents GA, CAT and EPI (mg/g) in bark samples of *S. asoca* from HEG, KOD and JAD.

Sample No.	HEG			KOD			JAD		
	GA	CAT	EPI	GA	CAT	EPI	GA	CAT	EPI
1	0.031±0.001	0.707±0.035	2.442±0.122	0.035±0.001	1.026±0.051	6.145±0.307	0.087±0.004	0.087±0.004	1.512±0.075
2	0.023±0.001	0.686±0.034	3.438±0.171	0.067±0.003	1.042±0.052	5.300±0.265	0.083±0.004	0.747±0.037	6.069±0.303
3	0.283±0.014	1.596±0.079	6.100±0.305	0.059±0.002	0.807±0.040	5.981±0.299	0.077±0.003	0.488±0.024	6.656±0.332
4	0.017±0.001	0.034±0.001	0.213±0.010	0.052±0.002	0.596±0.029	2.850±0.142	0.383±0.019	1.134±0.056	6.408±0.320
5	0.041±0.002	0.268±0.013	3.473±0.173	0.063±0.003	0.388±0.019	2.227±0.111	0.267±0.013	0.707±0.035	5.412±0.270
6	0.136±0.006	0.424±0.021	3.250±0.162	0.263±0.013	1.074±0.053	5.804±0.290	0.058±0.002	0.223±0.011	1.501±0.075
7	0.012±0.001	0.291±0.014	1.765±0.088	0.035±0.001	0.978±0.048	3.637±0.181	0.329±0.016	0.516±0.025	4.082±0.204
8	0.043±0.002	0.489±0.024	2.256±0.112	0.036±0.001	0.272±0.013	1.284±0.064	0.407±0.020	1.300±0.065	7.725±0.386
9	0.016±0.001	0.179±0.008	0.751±0.037	0.048±0.002	0.930±0.046	3.380±0.169	0.446±0.022	2.377±0.118	7.994±0.399
10	0.017±0.001	0.251±0.012	1.233±0.061	0.075±0.003	0.548±0.027	3.011±0.150	0.185±0.009	0.510±0.025	4.056±0.202

GA: gallic acid; CAT: catachin; EPI: epicatachin; HEG: Heggarni; KOD: Kodanamane; JAD: Jaddigadde.

**Table 2(C):** Contents GA, CAT and EPI (mg/g) in bark samples of *S. asoca* from BIL, BON and MAD.

Sample No.	BIL			BON			MAD		
	GA	CAT	EPI	GA	CAT	EPI	GA	CAT	EPI
1	0.047±0.002	0.785±0.039	4.375±0.218	0.111±0.005	0.481±0.0240	4.208±0.210	0.301±0.015	1.110±0.055	0.165±0.008
2	0.162±0.008	0.942±0.047	4.387±0.219	0.062±0.003	0.406±0.020	3.091±0.154	0.030±0.001	0.034±0.001	0.173±0.008
3	0.058±0.002	0.917±0.045	6.645±0.332	0.116±0.005	1.461±0.073	4.004±0.200	0.035±0.001	0.054±0.002	0.144±0.007
4	0.080±0.004	0.193±0.009	1.558±0.077	0.097±0.004	0.578±0.028	3.937±0.196	0.068±0.003	0.017±0.001	0.027±0.001
5	0.036±0.001	0.548±0.027	2.810±0.140	0.038±0.001	0.473±0.023	3.528±0.176	0.183±0.009	0.022±0.001	0.011±0.001
6	0.027±0.001	0.110±0.005	1.314±0.065	0.166±0.008	1.041±0.052	5.139±0.256	0.571±0.028	0.038±0.001	0.041±0.002
7	0.025±0.001	0.214±0.010	1.626±0.081	0.109±0.005	0.651±0.032	4.435±0.221	--	--	--
8	0.052±0.002	0.605±0.030	6.603±0.330	0.106±0.005	0.235±0.011	2.665±0.133	--	--	--
9	0.021±0.001	0.643±0.032	3.605±0.180	0.210±0.010	0.642±0.032	4.308±0.215	--	--	--
10	0.038±0.001	1.134±0.056	6.115±0.305	0.337±0.016	1.352±0.067	7.008±0.350	--	--	--

GA: gallic acid; CAT: catachin; EPI: epicatachin; BIL: Bilgi; BON: Bondla; Mad: Madgaon.

**Table 2(D):** Contents GA, CAT and EPI (mg/g) in bark samples of *S. asoca* from GHA and TIL.

Sample No.	GHA			TIL		
	GA	CAT	EPI	GA	CAT	EPI
1	0.068±0.003	0.864±0.043	2.348±0.117	0.007±0.001	0.326±0.016	3.202±0.160
2	0.089±0.004	0.789±0.039	4.651±0.232	0.016±0.001	0.409±0.020	3.269±0.163
3	0.174±0.008	1.739±0.086	4.011±0.200	0.113±0.005	0.487±0.024	3.900±0.195
4	0.101±0.005	0.798±0.039	3.566±0.178	0.195±0.009	0.518±0.025	5.482±0.274
5	0.107±0.005	0.585±0.029	2.740±0.137	0.074±0.003	0.433±0.021	3.088±0.154
6	ND	0.108±0.005	3.388±0.169	0.082±0.004	0.556±0.027	4.523±0.226
7	0.195±0.009	0.872±0.043	5.953±0.297	0.205±0.010	0.737±0.036	4.744±0.237
8	0.436±0.021	1.887±0.094	10.216±0.510	0.218±0.010	0.779±0.038	4.707±0.235
9	0.159±0.007	0.809±0.040	4.063±0.203	0.063±0.003	0.391±0.019	4.399±0.219
10	0.327±0.016	1.463±0.073	7.443±0.372	0.257±0.012	0.664±0.033	3.235±0.161

GA: gallic acid; CAT: catachin; EPI: epicatachin; ND: not detected; GHA: Ghativade; TIL: Tillari.

**Table 3(A):** Contents GA, CAT and EPI (mg/g) in leaf samples of *S. asoca* from AMG, DEV and THI.

Sample No.	AMG			DEV			THI		
	GA	CAT	EPI	GA	CAT	EPI	GA	CAT	EPI
11	0.154±0.007	0.802±0.040	0.303±0.015	0.859±0.042	0.046±0.002	0.049±0.002	0.965±0.048	1.005±0.050	0.168±0.008
12	0.063±0.003	0.272±0.013	0.115±0.005	1.815±0.090	0.118±0.005	0.115±0.005	1.203±0.060	0.545±0.027	0.448±0.022
13	0.334±0.016	0.597±0.029	0.194±0.009	1.365±0.068	0.115±0.005	0.054±0.002	0.026±0.001	0.005±0.001	0.009±0.001
14	0.038±0.001	0.234±0.011	0.074±0.003	1.464±0.073	0.114±0.005	0.016±0.001	0.711±0.035	0.031±0.001	0.096±0.004
15	0.174±0.008	0.704±0.035	0.832±0.041	0.956±0.047	0.056±0.002	0.051±0.002	0.593±0.029	0.242±0.012	0.630±0.031
16	0.060±0.003	0.042±0.002	0.039±0.001	1.045±0.052	0.052±0.002	0.030±0.001	10.018±0.500	0.503±0.025	2.160±0.108
17	0.114±0.005	0.232±0.011	0.166±0.008	1.559±0.077	0.054±0.002	0.031±0.001	1.143±0.057	0.318±0.015	0.438±0.021
18	0.082±0.004	0.384±0.019	0.403±0.020	1.138±0.056	0.272±0.013	0.413±0.020	0.036±0.001	0.009±0.001	0.047±0.002
19	0.079±0.003	0.073±0.003	0.035±0.001	0.639±0.031	0.032±0.001	0.290±0.014	0.863±0.043	0.470±0.023	0.179±0.008
20	0.150±0.007	0.335±0.016	1.099±0.054	0.786±0.039	0.042±0.002	0.009±0.001	0.345±0.017	0.086±0.004	0.020±0.001

GA: gallic acid; CAT: catachin; EPI: epicatachin; AMG: Amgaon; DEV: Devimane Ghat; THI: Thirthahalli.

**Table 3(B):** Contents GA, CAT and EPI (mg/g) in leaf samples of *S. asoca* from HEG, KOD and JAD.

Sample No.	HEG			KOD			JAD		
	GA	CAT	EPI	GA	CAT	EPI	GA	CAT	EPI
11	0.102±0.005	0.005±0.001	ND	1.599±0.079	1.461±0.073	2.182±0.109	0.263±0.013	0.093±0.004	1.072±0.053
12	1.199±0.059	0.103±0.005	2.048±0.102	1.288±0.064	0.726±0.036	1.215±0.060	2.863±0.143	0.037±0.001	1.881±0.094
13	2.271±0.113	0.026±0.001	0.206±0.010	0.805±0.040	1.141±0.057	1.708±0.085	2.795±0.139	0.166±0.008	0.878±0.043
14	0.492±0.024	0.013±0.001	0.025±0.001	0.522±0.026	0.309±0.015	1.468±0.073	1.075±0.053	0.256±0.012	0.474±0.023
15	1.318±0.065	0.030±0.001	0.059±0.002	0.799±0.039	0.550±0.027	0.157±0.007	1.183±0.059	ND	2.033±0.101
16	1.334±0.066	0.174±0.008	0.379±0.018	1.412±0.070	0.226±0.011	3.139±0.156	0.653±0.032	0.564±0.028	4.183±0.209
17	0.181±0.009	0.043±0.002	0.052±0.002	0.596±0.029	0.033±0.001	0.281±0.014	0.566±0.028	0.955±0.047	1.457±0.072
18	0.151±0.007	0.038±0.001	0.111±0.005	1.965±0.098	0.201±0.010	0.818±0.040	0.920±0.046	1.320±0.066	1.704±0.085
19	0.076±0.003	0.002±0.001	0.012±0.001	1.640±0.082	0.220±0.011	0.381±0.019	0.560±0.028	0.526±0.026	4.553±0.227
20	0.324±0.016	0.121±0.006	0.539±0.026	1.097±0.054	0.212±0.010	0.436±0.021	1.430±0.071	0.069±0.003	0.312±0.015

GA: gallic acid; CAT: catachin; EPI: epicatachin; ND: not detected; HEG: Heggarni; KOD: Kodanamane; JAD: Jaddigadde.

**Table 3(C):** Contents GA, CAT and EPI (mg/g) in leaf samples of *S. asoca* from BIL, BON and MAD.

Sample No.	BIL			BON			MAD		
	GA	CAT	EPI	GA	CAT	EPI	GA	CAT	EPI
11	0.326±0.016	0.011±0.001	0.010±0.001	1.188±0.059	0.631±0.031	0.610±0.030	3.613±0.180	0.971±0.048	1.752±0.087
12	0.380±0.019	0.017±0.001	0.035±0.001	1.223±0.061	0.784±0.039	2.441±0.122	0.246±0.012	0.054±0.002	0.007±0.001
13	1.125±0.056	0.352±0.017	1.369±0.068	0.471±0.023	0.083±0.004	0.018±0.001	0.115±0.005	0.025±0.001	0.007±0.001
14	0.605±0.030	0.252±0.012	0.423±0.021	1.248±0.062	0.573±0.028	0.898±0.044	0.556±0.027	0.350±0.017	0.039±0.001
15	1.017±0.050	0.517±0.025	1.523±0.076	0.990±0.049	0.334±0.016	1.004±0.050	0.377±0.018	0.187±0.009	0.083±0.004
16	0.548±0.027	0.174±0.008	0.073±0.003	0.908±0.045	0.600±0.030	1.614±0.080	0.422±0.021	0.118±0.005	0.069±0.003
17	0.766±0.038	0.344±0.017	0.693±0.034	1.137±0.056	0.377±0.018	1.070±0.053	--	--	--
18	0.635±0.031	0.243±0.012	0.290±0.014	0.772±0.038	0.375±0.018	0.201±0.010	--	--	--
19	0.467±0.023	ND	0.103±0.005	1.161±0.058	0.689±0.034	1.056±0.052	--	--	--
20	0.343±0.017	0.209±0.010	0.319±0.015	0.709±0.035	0.102±0.005	0.645±0.032	--	--	--

GA: gallic acid; CAT: catachin; EPI: epicatachin; ND: not detected; BIL: Bilgi; BON: Bondla; Mad: Madgaon.

**Table 3(D):** Contents GA, CAT and EPI (mg/g) in leaf samples of *S. asoca* from GHA and TIL.

Sample No.	GHA			TIL		
	GA	CAT	EPI	GA	CAT	EPI
11	1.187±0.059	0.266±0.013	0.889±0.044	0.805±0.040	0.348±0.017	1.210±0.060
12	0.661±0.033	0.547±0.027	1.481±0.074	0.764±0.038	0.280±0.014	1.010±0.050
13	0.849±0.042	0.318±0.015	1.326±0.066	0.631±0.031	0.392±0.019	3.404±0.170
14	0.893±0.044	0.574±0.028	0.311±0.015	0.623±0.031	0.444±0.022	1.117±0.055
15	0.727±0.036	0.418±0.020	1.026±0.051	0.829±0.041	0.320±0.016	4.382±0.219
16	1.194±0.059	0.112±0.005	1.492±0.074	0.965±0.048	0.426±0.021	5.594±0.279
17	0.831±0.041	0.359±0.017	0.493±0.024	0.308±0.015	0.078±0.003	0.340±0.017
18	1.074±0.053	0.395±0.019	1.679±0.083	0.696±0.034	0.634±0.031	0.576±0.028
19	0.359±0.017	0.832±0.041	0.924±0.046	0.748±0.037	0.428±0.021	2.572±0.128
20	0.558±0.027	0.900±0.045	1.413±0.070	1.901±0.095	0.423±0.021	0.473±0.023

GA: gallic acid; CAT: catachin; EPI: epicatachin; GHA: Ghativade; TIL: Tillari.

**Table 4:** Standard error of the mean (SEM) of chemical constituents of gallic acid (GA), catechin (CAT) and epicatechin (EPI) from 106 leaf samples of *S. asoca* populations.

State	Localities	GA	EPI	CAT
Bark				
Karnataka	AMG	0.002	0.011	0.008
	JAD	0.004	0.073	0.020
	DEV	0.002	0.046	0.010
	KOD	0.002	0.054	0.009
	HEG	0.002	0.053	0.014
	BIL	0.001	0.065	0.011
	THI	0.006	0.017	0.008
Maharashtra	TIL	0.002	0.026	0.004
	GHA	0.004	0.076	0.017
Goa	BON	0.002	0.037	0.013
	MAD	0.008	0.003	0.017
Leaf				
Karnataka	AMG	0.012	0.021	0.012
	JAD	0.028	0.045	0.014
	DEV	0.011	0.004	0.002
	KOD	0.015	0.030	0.014
	HEG	0.023	0.019	0.001
	BIL	0.008	0.017	0.005
	THI	0.094	0.020	0.010
Maharashtra	TIL	0.013	0.058	0.004
	GHA	0.008	0.014	0.007
Goa	BON	0.008	0.021	0.007
	MAD	0.054	0.028	0.014

**Table 5:** Total chemical constituent (mg/g) variation within the 11 populations of *S. asoca.*

Sl. No	Part	Min	Max	Mean	Std. Dev	95% CI		Median	IQ Range	
						Low	High		25^th^	75^th^
1	GA (Bark)	0.0	1.397	0.179	0.228	0.135	0.223	0.090	0.045	0.221
2	GA (Leaf)	0.0266	10.019	0.927	1.084	0.718	1.136	0.769	0.380	1.167
3	GA (All)	0.0	10.019	0.553	0.866	0.436	0.671	0.317	0.078	0.806
4	EPI (Bark)	0.011	10.217	3.022	2.265	2.586	3.458	3.050	1.220	4.409
5	EPI (Leaf)	0.0	5.594	0.853	1.069	0.647	1.059	0.438	0.081	1.244
6	EPI (All)	0.0	10.217	1.937	2.075	1.656	2.218	1.225	0.221	3.265
7	CAT (Bark)	0.0	2.377	0.597	0.444	0.512	0.683	0.514	0.265	0.807
8	CAT (Leaf)	0.0	1.461	0.324	0.304	0.265	0.382	0.255	0.072	0.478
9	CAT (All)	0.0	2.377	0.461	0.404	0.406	0.515	0.381	0.134	0.649

GA: gallic acid; EPI: epicatechin; CAT: catechin; CI: Confidence Interval; IQ: Intelligence Quotient.

**Table 6:** ISSR markers associated with high (≥75^th^ percentile) concentration of phytochemicals in 11 *S. asoca* populations.

Bark									
	GA			EPI			CAT		
	β	OR	p	β	OR	p	β	OR	p
L14				-1.19	0.31	**0.024**	-1.26	0.28	**0.016**
L22	1.64	5.17	**0.007**						
L29	2.26	9.60	**0.001**						
L33				1.76	5.80	**0.002**			
L42				1.21	3.35	**0.021**			
L44	1.03	2.80	**0.048**						
L54							1.51	4.54	**0.006**
L74							1.95	6.99	**0.006**
Leaf									
L06							3.341	31.231	**0.002**
L08				1.350	3.857	**0.006**			
L09							-29.315	0.000	1.000
L14	-1.924	0.146	**0.001**						
L27				1.213	3.365	**0.018**			
L54	1.137	3.118	**0.024**

**Table 7:** Prediction accuracy of models for phytochemical concentrations (≥75^th^ percentile = high, else = Low) in 11 *S. asoca* populations.

Bark									
	Low			High			Total		
	N	Predicted	(%)	N	Predicted	(%)	N	Predicted	(%)
GA	78	75	96.2	28	10	35.7	106	85	80.2
EPI	79	75	94.9	27	9	33.3	106	84	79.2
CA	79	67	84.8	27	15	55.6	106	82	77.4
Leaf									
GA	79	71	89.9	27	11	40.7	106	82	77.4
EPI	79	75	94.9	27	11	40.7	106	86	81.1
CA	98	98	100.0	8	0	0.0	106	98	92.5

## 2 Experimental design, materials, and methods

### 2.1 Plant material collection

The plant materials were collected from Western Ghats regions of Karnataka, Maharashtra and Goa states of India [1]. Total 106 accessions of 11 population of *Saraca asoca* (Roxb.) De Wilde leaf and bark were collected and authenticated by taxonomist. Voucher specimen has been deposited at ICMR-National Institute of Traditional Medicine with Voucher Number: RMRC 997. The identity of the species was also authenticated by amplification and sequencing of *matK* region of the voucher specimen [1]. Each leaf sample from all accessions were stored at −80 °C for DNA extraction. Leaf and bark samples were shade dried before performing extraction process.

### 2.2 Molecular analysis

#### 2.2.1 DNA extraction

DNA extraction was performed using modified CTAB method by using 1g of all 106 accessions of leaf samples [3]. The isolated DNA were electrophoresed using 1% agarose gels, stained with GelRed for detection of DNA and ensuring acceptable quality, whereas quantification was performed using Nanodrop spectrophotometer (JH BIO) [1].

#### 2.2.2 PCR amplification and its characterization

PCR was performed with 7 primers (Table 1) on the plant DNA samples. Previously published standard PCR conditions were maintained for amplification of extracted DNA samples [1], [4]. The PCR products were separated by electrophoresis in a 1.5% agarose gel under 80 V electrical current, stained with GelRed, and visualized using gel documentation system (Syngene, UK). The banding pattern of the accessions were scored as, presence (1) or absence (0) and binary matrix was constructed [1], [5]. The number of polymorphic characters with each primer like Polymorphic Information Content (PIC) and Marker Index (MI) were recorded [1], [6]. Relationship between geographical and genetic distance and analysis of molecular variance (AMOVA) were carried out using GenAlEx 6.5 [7], [8]. Population genetic structure was assayed using STRUCTURE version 2.3.1 with admixture model to determine the number of sub-populations [1], [9], [10], [11].

### 2.3 Phytochemical analysis

#### 2.3.1 Extract preparation

Extraction was carried out using 5g shade dried powdered samples (leaf and bark) in 50 mL petroleum ether for 12–16 h. This procedure was repeated twice and the pooled extracts were evaporated to dryness. Further, 50 mL of methanol: water (70:30) was added into this and the mixture was kept for 12–16h, followed by 15 min sonication [1]. This extraction was repeated two times to collect a total of 150 mL of extract which was further filtered and evaporated to dryness [1].

#### 2.3.2 EPI, GA and CAT concentrations and their analysis

The leaf and bark samples from all accessions were processed by HPLC based method for quantitation of gallic acid (GA), epicatechnin (EPI) and catechin (CAT) [1]. The GA, EPI and CAT concentration (in mg/g) of all 106 accessions were summarised in terms of range (minimum and maximum), standard deviation, mean, 95% confidence interval, median and inter-quartile range. The distribution of *S. asoca* along with those of common adulterant/substituent (*P. longifolia*) obtained from previous study [2] were used to construct dot-plots with median values. The GA, CAT and EPI concentrations were used to construct receiver operating characteristic curves for both bark, leaf and all with false positivity (1- specificity) on the X-axis and sensitivity on the Y-axis (Fig. 5). Considering GA, EPI and CAT as dependent variables and bands as independent variables a multiple logistic regression was performed (Table 6). Table 7 depicts the prediction of high and low concentrations and overall prediction ability for each model. These studies were performed separately for leaf and bark samples of *S. asoca*. BioVinci version 1.1.0 for Windows (BioTuring Inc., San Diego California USA) was used to perform Principal Component Analysis (PCA) [1].
